# *Origanum vulgare* L. essential oil inhibits virulence patterns of *Candida* spp. and potentiates the effects of fluconazole and nystatin in vitro

**DOI:** 10.1186/s12906-022-03518-z

**Published:** 2022-02-09

**Authors:** Camila Cid-Chevecich, Andrea Müller-Sepúlveda, José Antonio Jara, Rodrigo López-Muñoz, Rocío Santander, Mauricio Budini, Alejandro Escobar, Raúl Quijada, Alfredo Criollo, Mario Díaz-Dosque, Alfredo Molina-Berríos

**Affiliations:** 1 Laboratory of Pharmacology, Institute for Research in Dental Sciences (ICOD), Faculty of Dentistry, University of Chile, Olivos 943, Independencia, Santiago, Chile; 2 Institute of Agrifood, Animals and Environmental Sciences, Universidad de O’Higgins, San Fernando, Chile; 3 Instituto de Farmacología y Morfofisiología, Facultad de Ciencias Veterinarias, Universidad Austral de Chile, Valdivia, Chile; 4 Department of Environmental Sciences, Faculty of Chemistry and Biology, Universidad de Santiago de Chile, Santiago, Chile; 5 Faculty of Physical and Mathematical Sciences, Universidad de Chile, Santiago, Chile

**Keywords:** *Candida albicans*, Essential oils, Biofilms, *Origanum vulgare*

## Abstract

**Background:**

Recurrence and resistance of *Candida* spp. infections is associated with the ability of these microorganisms to present several virulence patterns such as morphogenesis, adhesion, and biofilm formation. In the search for agents with antivirulence activity, essential oils could represent a strategy to act against biofilms and to potentiate antifungal drugs.

**Objective:**

To evaluate the antivirulence effect of *Origanum vulgare* L. essential oil (O-EO) against *Candida* spp. and to potentiate the effect of fluconazole and nystatin.

**Methods:**

The effect of O-EO was evaluated on ATCC reference strains of *C. albicans* and non-*albicans Candida* species. Minimum inhibitory concentration (MIC) was determined through broth microdilution assay. Adhesion to microplates was determined by crystal violet (CV) assay. An adapted scratch assay in 24-well was used to determine the effect of essential oil on biofilms proliferation. Viability of biofilms was evaluated by MTT reduction assay and through a checkerboard assay we determined if O-EO could act synergistically with fluconazole and nystatin.

**Results:**

MIC for *C. albicans* ATCC-90029 and ATCC-10231 was 0.01 mg/L and 0.97 mg/L, respectively. For non-albicans *Candida* strains MIC values were 2.6 mg/L for *C. dubliniensis* ATCC-CD36 and 5.3 mg/L for *C. krusei* ATCC-6258. By using these concentrations, O-EO inhibited morphogenesis, adhesion, and proliferation at least by 50% for the strains assayed. In formed biofilms O-EO decreased viability in ATCC 90029 and ATCC 10231 strains (IC50 7.4 and 2.8 mg/L respectively). Finally, we show that O-EO interacted synergistically with fluconazole and nystatin.

**Conclusions:**

This study demonstrate that O-EO could be considered to improve the antifungal treatment against *Candida* spp.

**Supplementary Information:**

The online version contains supplementary material available at 10.1186/s12906-022-03518-z.

## Background

Biofilms are associated with over 80% of human microbial infections [1]. *Candida albicans*-related infections are no exception, as the ability to form biofilms is one of the major virulence attributes of this fungus. *C. albicans* is involved in invasive candidiasis, vulvovaginitis, esophageal candidiasis and oral candidiasis [2, 3]. Recent global reports estimate that yeasts from the genus *Candida* are responsible for 400,000 new cases of life-threatening candidiasis each year, with mortality rates of up to 40–60%, even with antifungal treatment [4, 5]. This statistic can be explained in part by increasing resistance to antifungal drugs over recent years, a problem aggravated by the slow pace with which new antifungals have been developed [6]. Furthermore, there is a growing role of non-*albicans Candida* species as causative agents of emerging infections. These species are often more resistant than *C. albicans* strains, making the search for new pharmacological strategies imperative [7, 8].

*Candida* spp. biofilms are formed when a cell population adheres to an abiotic or biological surface and produces an extracellular matrix (ECM), which encloses and protects the microorganisms from the host immune system and external factors such as antifungals [9]. Among all *Candida* spp., *C. albicans* is the most virulent and the predominant yeast in human infections, responsible for about 50% of human candidiasis cases [10]. *C. albicans* biofilms are extremely resistant to conventional antifungals and represent one of the main causes of treatment failure and mortality in systemic candidiasis [11, 12]. This is due to the ability of *Candida* spp. to form biofilms on various medical devices, such as indwelling catheters, dentures, prosthetic heart valvules, among others [13].

Host cells and fungi share metabolic features, which makes it challenging to develop drugs with both pathogen specificity and low toxicity to mammal cells and normal microbiota [6]. To overcome these problems, drugs targeting pathogen-specific virulence factors have emerged as a new approach [9]. In *C. albicans* biofilm formation, the first apparent virulence feature is adhesion, driven by expression of several surface adhesins proteins [14]. During the filamentation process, *C. albicans* cells transition from an ovoid yeast to an elongated hyphal form. This change allows the fungus to colonize tissues, express drug-resistance proteins and begin biofilm formation [15]. Several studies have shown that the virulence of *C. albicans* is decreased or eliminated if morphogenic ability is impaired [15, 16].

The search for substances that target virulence phenotypes as emerged as a new approach to decrease the pathogenicity of biofilms and to potentiate the effects of conventional antifungals. This strategy may ultimately serve to decrease antifungal resistance, minimize the dosages required and reduce drug toxicity [9]. In this context, natural products such as essential oils have emerged as a novel strategy to inhibit biofilms and potentiate conventional antifungals against *Candida* spp. [17–20]. We recently evaluated the effect of *Lavandula dentata* L. essential oil and reported a high antibiofilm effect against *C. albicans* strains [21]. Essential oil from *Origanum vulgare* L. has gained attention in the past years, demonstrating impressive antioxidant, anti-inflammatory and antimicrobial effects [22, 23]. Although this essential oil has recognized anticandidal activity, there are no reports indicating whether *O. vulgare* could be used to inhibit virulence patterns in *C. albicans* and non-*albicans* strains. Furthermore, no prior studies have explored the potential of this oil to potentiate conventional antifungals against established *C. albicans* biofilms. Therefore, we investigated the effects of *O. vulgare* essential oil on phenotypic virulence features of Candida spp. as well as its effectiveness against biofilms in combination with fluconazole and nystatin.

## Materials and methods

### Plant material and essential oil preparation

The aerial parts of *O. vulgare* were collected during spring flowering in Chicauma, Lampa, Metropolitan Region, Chile (33°14′23″S, 70°54′27″W). The specimen was identified by the taxonomist Alicia Marticorena (MSc) at the Herbario CONC, Universidad de Concepción, Chile (herbarium registration number CONC 191040). A copy of the certificate is available in the Supporting Information. Essential oil was extracted from 300 g of fresh leaves by steam distillation for 3 h, yielding an average of 2.5 mL per extraction cycle. The density of the essential oil was 0.920 g/mL, calculated as the ratio of the mass of 1 mL of essential oil and the mass of 1 mL of distilled water at 20 °C. The collected essential oil was stored at − 20 °C until use.

### Gas chromatography-mass spectrometry (GC-MS) analysis of essential oil

The *O. vulgare* essential oil (O-EO) was diluted with dichloromethane, and 1 μL of the sample was analyzed using a GC-MS/MS, Thermo Scientific (GC: model Trace 1300 and MS: model TSQ8000Evo) operating in EI mode at 70 eV, equipped with a splitless injector (250 °C). The transfer line temperature was 250 °C. Helium was used as a carrier gas at a rate of 1.2 mL/min, and the capillary column used was a Rtx-5 ms (60 m × 0.25 mm i.d., film thickness 0.25 μm). The temperature program was 40 °C (5 min) to 300 °C (5 min) at a rate of 5 °C/min. The chemical composition of the oil was identified by comparing its spectra with an NIST14 library and confirmed by contrasting the retention indices with data published in other studies.

### Minimum inhibitory concentration determination (MIC)

The MIC was determined according to the EUCAST method for susceptibility testing of yeasts (E.DEF 7.3.2, 2020), defined as the concentration able to inhibit the growth of planktonic yeasts by 50% [24]. To perform this assay, 0.5 × 10^5^ UFC/mL was added to each well on a 96-well polystyrene plate (Falcon) in RPMI-1640 (Sigma-Aldrich). Afterwards, O-EO was added at various concentrations (0.002–10 μg/mL) and incubated for 24 h at 37 °C. The essential oil was first diluted in DMSO and subsequently in RPMI to obtain a final DMSO concentration of 0.5–1.0% in each well. Growth inhibition was determined by measuring optical density at 450 nm on a 96-well plate reader (Infinite F50, Tecan). Fluconazole and nystatin were added at concentrations of 0.1–300 mg/L and 0.01–10 mg/L, respectively. Negative controls contained only DMSO. We used the MIC values obtained from planktonic cells as a reference for the remaining assays because the sessile cells could expect to be exposed to these concentrations under pharmacological treatment.

### Adhesion

The effects of the various treatments on the adhesion process were evaluated as previously described [21]. Cells (1 × 10^5^ UFC) were added to each well on a 96-well polystyrene plate in the presence or absence of O-EO, fluconazole or nystatin at the respective MIC. The plates were incubated at 37 °C in RPMI-1640 with fetal bovine serum (FBS) (10% v/v) for 4 h (initial adherence) without shaking. After incubation, non-adherent cells were discarded. Next, 100 μL of 0.1% CV aqueous solution was added to each well and the mixture incubated for 5 min at room temperature. The CV solution containing non-adhered cells was discarded, and the adhered cells were washed four times with phosphate buffer saline (PBS). Finally, 200 μL of acetic acid (10% v/v) was added to each well, and the supernatant was read at 595 nm on a 96-well plate reader (Infinite F50, Tecan). Results were expressed as the percentage of adhesion relative to the control group (DMSO).

### Morphogenesis

Cells (1 × 10^5^ UFC) were added to 1.5-mL microcentrifuge tubes and incubated for 5 h with agitation (125 rpm) at 37 °C in RPMI-1640 medium with FBS (10% v/v) (Gibco) in the presence or absence of essential oil, fluconazole or nystatin at the respective MIC. After incubation, the percentage of filamentation was calculated as the number of filamentous cells divided by the total number of cells observed by light microscopy [21]. At least 10 microscopic fields (approximately 200 cells) were observed under the microscope (40× magnification). The cells were categorized as a) non-filamentous (less than three joined cells) or b) filamentous cells, with true hyphae or pseudohyphae.

### Biofilm scratch assay

Biofilms were obtained by seeding 1 mL of a standardized suspension (1 × 10^6^ cells/mL) and 1 mL of RPMI-1640 supplemented with FBS (10% v/v) into each well on a 12-well plate for 24 h at 37 °C. After this period, the supernatant was discarded, and scratch wounds were made by scraping the cells with a sterilized 1-mL pipette tip (T0). The wells were then replenished with the same medium containing various concentrations of O-EO, fluconazole or nystatin (_1/2_MIC, MIC and 2xMIC). After 24 h, the wells were washed three times with PBS then stained and fixed with CV in methanol (0.5%) for 5 min and washed again four times with PBS (T1). Scratched areas were observed under light microscopy at T0 and T1. At least three random fields in each well in triplicate were analyzed using Fiji processing software (https://imagej.net/Fiji). Results were expressed as the percentage of biofilm scratch closure [21].

### Planktonic and biofilm viability assay

Viability was quantified using the MTT (Life Technologies) reduction assay, essentially as described by Barros Cota et al., 2021 [25]. For the planktonic cultures, 100 μL of a standardized suspension (1 × 10^6^ cells/mL) was added to each well on a 96-well polystyrene plate in RPMI-1640 medium and incubated for 24 h at 28 °C in the presence or absence of each treatment at the same concentrations used for MIC determination. For the biofilm viability assays, 100 μL of a standardized suspension (1 × 10^6^ cells/mL) and 100 μL of RPMI-1640 medium supplemented with FBS (10% v/v) were added to each well on a 96-well polystyrene plate for 24 h at 37 °C. After this period, the biofilms were washed three times with 100 μL of sterile PBS to remove nonadherent cells. Biofilms were then treated with O-EO at various concentrations (0.5–20 mg/L) for 24 h at 37 °C. Fluconazole and nystatin were added at concentrations of 0.01–300 mg/L and 0.1–100 mg/L, respectively. After this period, the biofilms were washed twice with PBS 1X, and then 100 μL of 0.5 mg/mL MTT solution was added to each well. After 4 h of incubation, MTT was removed, and formazan crystals were dissolved in 40 μL of DMSO. Absorbance was measured at 570 nm with a microplate ELISA reader (Infinite F50®Tecan Group Ltd., Swiss). IC_50_ values (concentrations inhibiting 50% of viability) were determined for each drug alone from the respective dose-response curves.

### Checkerboard assay

Combination studies with fluconazole/nystatin and O-EO were performed using microdilution checkerboard assay. In brief, we created a two-dimensional checkerboard on a 96-well plate with serial dilutions of each treatment. We analyzed for synergistic, antagonistic or additive interactions using the Loewe model. Viability was evaluated by MTT reduction as described above, and data were analyzed using COMBENEFIT software [26].

### Statistical analysis

Results were expressed as mean ± SD and analyzed using one- or two-way ANOVA (GraphPad Prism 6.0). Results were considered statistically significant for *p*-values < 0.05. All experiments were performed at least in triplicate on three independent days (*n* = 3).

## Results

### Main components present in O-EO

Chemical characterization of the O-EO essential oil was performed through gas chromatography-mass spectrometry (GC-MS). As shown in Table 1, oxygenated monoterpenes were the main constituents of the essential oil, accounting for 64.13% of the sample, followed by monoterpene hydrocarbons (26.59%) and sesquiterpenes (5.12%). The four main components of the essential oil analyzed were *cis-*sabinene hydrate (16.72%), followed by 4-terpineol (13.57%), thymol (14.20%) and γ-terpinene (11.66%). The chromatogram of the sample is available as supplementary Fig. 1.

**Table 1:** Compounds detected in *O. vulgare* essential oil

N°	Compound	% area	RI^**a**^	Identification
**Monoterpenes hydrocarbons (26.59%)**				
1	α-thujene	0.65	944.2	MS, RI^b^
2	α-pinene	0.16	953.9	MS, RI^b^
3	sabinene	2.84	991.2	MS, RI^b^
4	β-myrcene	1.07	1005.9	MS, RI^b^, Co-I
5	α-phellandrene	0.48	1024.6	MS, RI^b^
6	α-terpinolene	5.02	1037.5	MS, RI^c^
7	p-Cymene	2.20	1045.8	MS, RI^b^
8	3-carene	0.41	1055.8	MS, RI^b^
9	**γ-terpinene**	**11.66**	1080.3	MS, RI^b^
10	α-terpinolene	1.87	1110.4	MS, RI^b^, Co-I
11	*trans*-allo-ocimene	0.23	1150.5	MS, RI^b^
**Oxygenated Monoterpenes (64.13%)**				
12	4-thujanol	3.01	1089.2	MS, RI^b^,
13	linalool	0.55	1119.6	MS, RI^b^
14	***cis*-sabinene hydrate**	**16.72**	1124.0	MS, RI^b^
15	*cis*-β-terpineol	0.02	1128.7	MS, RI^b^, Co-I
16	*cis*-2-menthenol	1.21	1147.6	MS, RI^b^
17	*trans*-2-menthenol	0.78	1165.7	MS, RI^b^
18	pinocarvone	0.02	1192.0	MS, RI^b^
19	*endo*-borneol	0.28	1194.8	MS, RI^b^
20	**4-terpineol**	**13.57**	1205.5	MS, RI^b^, Co-I
21	α-terpineol	3.13	1218.3	MS, RI^b^, Co-I
22	*trans*-piperitol	0.30	1224.1	MS, RI^b^
23	*cis*-piperitol	0.38	1236.0	MS, RI^b^
24	methyl carvacrol	3.17	1269.9	MS, RI^b^
25	linalyl acetate	3.99	1277.1	MS, RI^b^
26	**thymol**	**14.20**	1314.5	MS, RI^b^
27	carvacrol	2.64	1324.0	MS, RI^b^
28	geranyl acetate	0.16	1406.2	MS, RI^b^
**Sesquiterpenes (5.12%)**				
29	caryophyllene	3.56	1465.2	MS, RI^b^
30	α-humulene	0.32	1499.2	MS, RI^b^
31	bicyclogermacrene	1.24	1544.6	MS, RI^b^
**Oxygenated Sesquiterpenes (0.47%)**				
32	neryl acetate	0.08	1387.5	MS, RI^b^
33	spathulenol	0.27	1631.7	MS, RI^b^
34	caryophyllene oxide	0.12	1640.5	MS, RI^b^
**Others (2.55%)**				
35	Cyclohexane, 1-methylene-4-(1-methylethenyl)-	1.74	1051.9	MS, RI^b^
36	benzene, 2-methoxy-4-methyl-1-(1-methylethyl)-	0.81	1259.9	MS, RI^b^
	**Percentage of identified molecules**	**98.86**		

^a^: Experimental retention index for non-polar column; ^b^: bibliographic retention index for non-polar column, *MS* Mass spectra, *Co-I* Co-injection of standard

### Antifungal activity of O-EO against *Candida albicans* and non-*albicans* strains

**Fig. 1:**
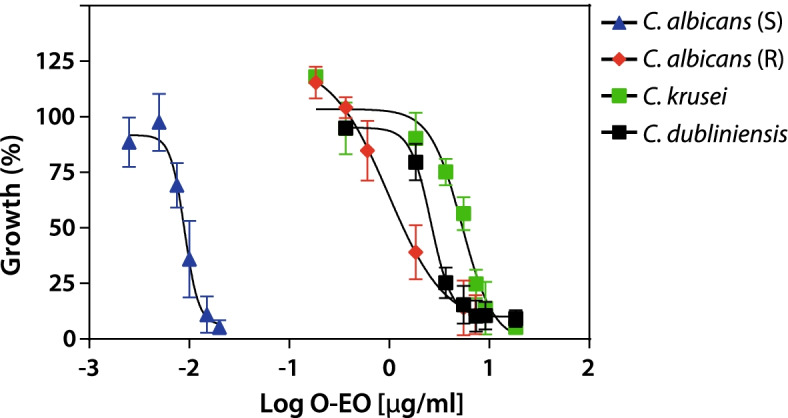
Antifungal activity of *Origanum* essential oil against *Candida albicans* and non-*albicans* strains. Dose-response curves were obtained from the culture of planktonic yeasts exposed to different concentrations of *Origanum essential oil* for 24 h at 37 °C. Curves represent the mean of at least three independent experiments

After characterizing the essential oil, we evaluated the antifungal effects of O-EO against *C. albicans* and non-*albicans Candida* reference strains. Using the broth microdilution method, we obtained the minimum inhibitory concentration (MIC) of fluconazole, nystatin and O-EO for each strain. Under our experimental conditions, the MIC value corresponds to the concentration able to inhibit the growth of the strains assayed by 50% (IC_50_). As shown in Table 2, *C. albicans* strains ATCC 90029 and ATCC 10231 were the most susceptible, with MIC values of 0.01 μg/mL and 0.97 μg/mL, respectively. For the non-*albicans* strains *C. krusei* and *C. dubliniensis*, the MIC values were 5.33 μg/mL and 2.61 μg/mL, respectively. Figure 1 shows representative dose-response curves for all strains assayed. As indicated, ATCC 90029 was the strain most susceptible to the actions of O-EO.

**Table 2:** Minimum Inhibitory Concentration of *O. vulgare* essential oil against *C. albicans* and non-*albicans*

	Minimum inhibitory concentration (mg/L)			
	***C. albicans*** ATCC 90029	***C. albicans*** ATCC 10231	***C. krusei*** ATCC 6258	***C. dubliniensis*** CD36
**Fluconazole**	0.76 ± 0.1	256 ± 10	44.52 ± 1.3	1.10 ± 0.1
**Nystatin**	0.33 ± 0.10	0.48 ± 0.10	1.40 ± 0.40	3.52 ± 0.30
**O-EO**	0.01 ± 2.3 × 10^−4^	0.97 ± 0.32	5.33 ± 0.87	2.61 ± 0.26

Results are expressed as mean (mg/L) + SD of at least three independent experiments. *O-EO Origanum* essential oil

### *O. vulgare* essential oil inhibits virulence factors in *Candida* spp.

#### Morphogenesis

**Fig. 2:**
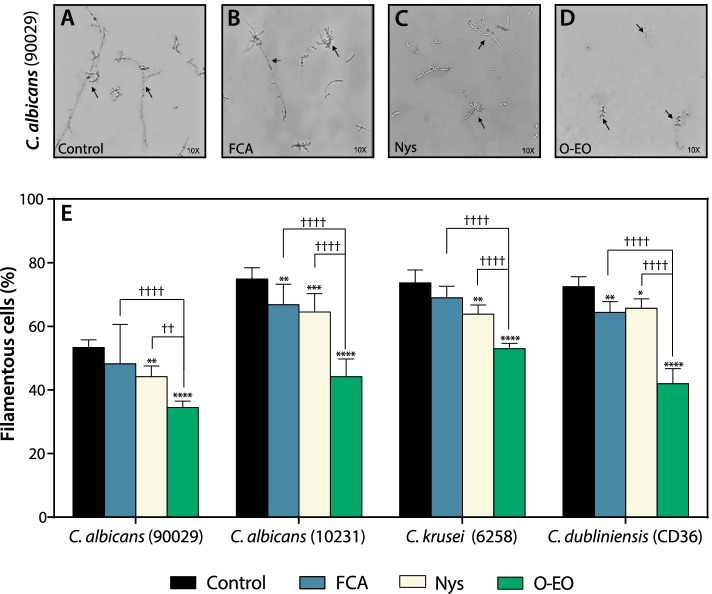
Origanum essential oil inhibits filamentation of *C. albicans* and non-*albicans* strains. Filamentation process was induced in absence and presence of the different treatments at their minimum inhibitory concentration (MIC) obtained previously. Then cells were observed by light microscopy. **A** Images showing the effect of fluconazole (FCA), nystatin (Nys) and *Origanum* essential oil (O-EO) on *C. albicans* ATCC 90029 strain. Arrows indicate the representative cell types observed which each treatment. **B** Quantification of filamentous cells. Bars represent the mean + SD of the percentage of filamentous cells relative to the total number of cells for each strain. *p < 0.1, **p < 0.01, ***p < 0.001, ****p < 0.0001 with respect to control (vehicle, DMSO); ††p < 0.01, ††††p < 0.0001 with respect to the indicated bar (two-way ANOVA). FCA: fluconazole, Nys: nystatin, O-EO: *Origanum* essential oil

Morphologic transition is a major virulence feature of *Candida* spp., with cells shifting from an ovoid shape to an elongated form with hyphae or pseudohyphae. Therefore, we evaluated whether O-EO could inhibit the *Candida* spp. filamentation process. We assessed filamentation in the presence or absence of O-EO, fluconazole and nystatin using the MIC obtained previously in the susceptibility assay. After inducing filamentation, cells were observed through a light microscope and counted using a Neubauer chamber to obtain the percentage of filamentous cells with respect to the untreated control for each strain. Figure 2 shows representative images of our experimental approach for the *C. albicans* ATCC 90029 strain and the effect of each treatment on the presence of filamentous cells. Control and fluconazole-treated cells showed long hyphae, while nystatin and O-EO decreased the both the number and size of elongated cells (Fig. 2 A-D). Figure 2E shows that filamentation of planktonic cells varied from ~ 57% in the most susceptible *C. albicans* strain (90029) to ~ 70–75% in the remaining strains assayed in the absence of treatment (Fig. 2E, black bars). Fluconazole treatment decreased filamentation by 11% in both *C. albicans* ATCC 10231 and *C. dubliniensis* ATCC CD36 (blue bars). The effect of nystatin on ATCC 10231 and ATCC CD36 was similar to that of fluconazole. Nystatin also decreased filamentation in the *C. albicans* ATCC 90029 strain by ~ 17% and in the *C. krusei* ATCC 6258 strain by 13% (light yellow bars). The effect of O-EO was significantly higher than that of fluconazole or nystatin. In the *C. albicans* ATCC 90029 and ATCC 10231 strains, treatment inhibited filamentation by 35 and 41%, respectively. In the non-a*lbicans Candida* strains, O-EO treatment decreased filamentation by 28% in *C. krusei a*nd 42% in *C. dubliniensis* (Fig. 2E, green bars).

Adhesion is a crucial step for biofilm formation in *Candida* spp. Out next step, then, was to evaluate whether O-EO could affect this process. Under our experimental conditions, non-*albicans* strains ATCC 6258 and CD36 failed to achieve significant adhesion to 96-well polystyrene plates. We therefore continued adhesion and biofilm formation experiments using only the *C. albicans* ATCC 90029 and ATCC 10231 strains.

### Adhesion

**Fig. 3:**
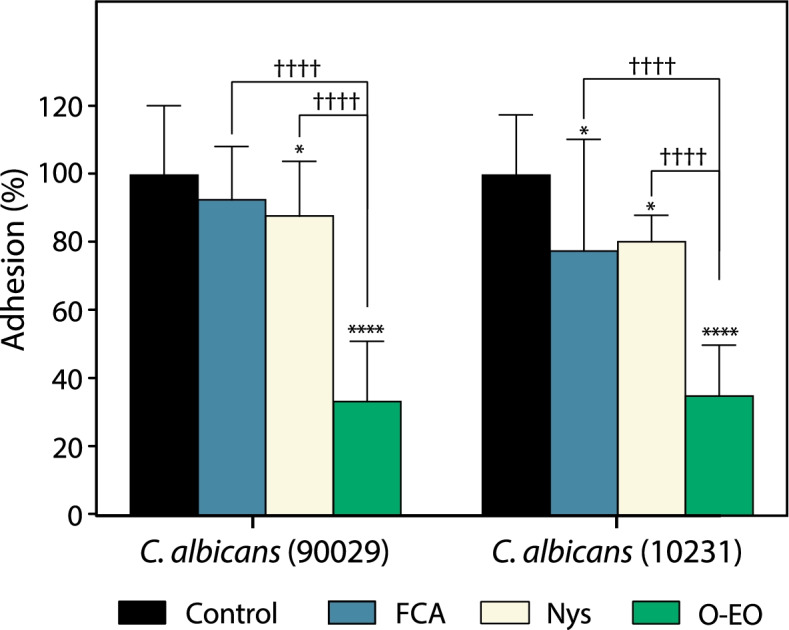
Inhibition of adhesion of *C. albicans* strains by *Origanum vulgare* essential oil. Cells were incubated without agitation for 4 h at 37 °C in presence or absence of treatments at the minimum inhibitory concentration (MIC) obtained previously according to methods. Then, non-attached cells were discarded, and percentage of adhesion was obtained considering untreated control as 100%. Bars represent the mean + SD of at least three independent experiments. *p < 0.1, ****p < 0.0001 with respect to control (vehicle, DMSO); ††††p < 0.0001 with respect to the indicated bar (two-way ANOVA). FCA: fluconazole, Nys: nystatin, O-EO: *Origanum* essential oil

In the presence of fluconazole, the adhesion decreased only in the ATCC10231 strain (Fig. 3). On the other hand, O-EO inhibited adhesion in both strains by approximately 60%. This effect was significantly greater than that exerted by nystatin (*p* < 0.0001).

**Fig. 4:**
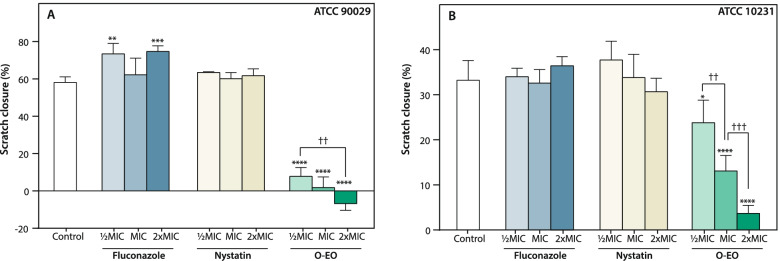
*Origanum vulgare* essential oil inhibits biofilm formation. Biofilm scratch assay. 24 h formed biofilms were scratched (T0) and then incubated for additional 24 h in absence (control, DMSO) or presence of treatments at different concentrations (MIC, _1/2_MIC, 2xMIC). After this period, scratched area was quantified and compared with T0. Bars represent the quantification of the healing of the wound area (mean + SD, *n* = 3), expressed as the percentage relative to T0. *p < 0.1, **p < 0.01, ***p < 0.001, ****p < 0.0001 with respect to control (vehicle, DMSO); ††p < 0.01, †††p < 0.001 with respect to the indicated bar (two-way ANOVA). O-EO: *Origanum* essential oil

To further evaluate the effect of O-EO as an antibiofilm agent, we performed experiments to evaluate its effects on pre-formed biofilms. First, we performed an adaptation of the classic cell culture scratch assay. As mentioned in the Methods section, we created biofilms on flat-bottom 12-well plates and then made a scratch wound by scraping the cells with a pipette tip. The biofilms were then incubated for additional 24 h in the presence or absence of various concentrations of the drugs, using the MIC values obtained for planktonic cells as the reference (1_/2_MIC, MIC and 2xMIC). Our objective was to evaluate whether, at these concentrations, O-EO could inhibit biofilm proliferation and prevent recolonization of the scratched biofilm areas. We calculated the initial area of the scratch (T0) and the area 24 h after treatment (T1), expressing the results as the percentage of scratch closure. Figure 4 shows that neither fluconazole nor nystatin was able to inhibit biofilm recovery in either strain assayed. In fact, biofilm proliferation increased in the presence of fluconazole in the ATCC 90029 strain (Fig. 4A). On the other hand, O-EO decreased proliferation in both strains in a concentration-dependent manner. Notably, in the ATCC 90029 strain, the area of the scratch wound actually increased with respect to the initial area at the highest concentration assayed (106% of inhibition, *p* < 0.0001).

### O-EO inhibits the viability of biofilms and potentiates the effects of fluconazole and nystatin

After observing the effect of O-EO on the initial biofilm formation steps (adhesion, morphogenesis and proliferation), we evaluated the effect of O-EO on the viability of formed biofilms. Using an MTT reduction viability assay, we constructed dose-response curves for fluconazole, nystatin and O-EO to identify the concentration that inhibited 50% of biofilm viability (IC_50_) (Table 3). Contrary to the results observed for planktonic cells, fluconazole was not able to affect the viability of biofilms, even at the highest dose tested (300 mg/L). Nystatin showed IC_50_ values of 12.7 mg/L for the *C. albicans* ATCC 90029 strain and 15.2 mg/L for ATCC 10231. The effect of O-EO was similar to that of nystatin against the ATCC 90029 strain biofilms (*p* > 0.05), but the IC_50_ value was significantly lower than that of nystatin for ATCC 10231 (2.8 mg/L O-EO vs. 15.2 mg/L nystatin, *p* < 0.0001).

**Table 3:** IC_50_ values of *O. vulgare* essential oil against *C. albicans* biofilms

	***C. albicans*** ATCC 90029	***C. albicans*** ATCC 10231
**Fluconazole**	ND	ND
**Nystatin**	12.7 ± 5.2	15.2 ± 2.6
**O-EO**	7.45 ± 2.8	2.8 ± 1.1 ****

Results are expressed as mean (mg/L) + SD of at least three independent experiments. O-EO*: O. vulgare* essential oil. ND: not detected. ****p < 0.0001 respect to nystatin IC_50_ (ANOVA)

**Fig. 5:**
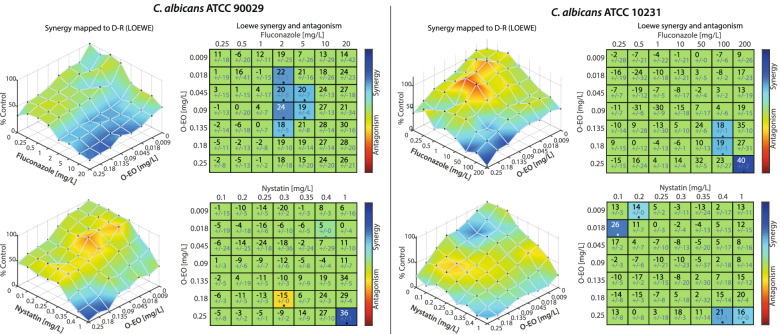
Effect of *Origanum vulgare* essential oil combined with fluconazole and nystatin on the viability of planktonic *Candida albicans* ATCC 90029 and ATCC 10231 strains. The effects of the combination of O-EO with fluconazole (upper row) and nystatin (lower row) on cell viability were measured using the MTT reduction assay in two *C. albicans strains*: ATCC 90029 (left panels) and ATCC 10231 (right panels). Under planktonic culture conditions yeasts were exposed to the different drugs for 24 h. Seven concentrations were mixed in every possible combination to obtain an effect matrix plotted with the Combenefit software. The graphs at the left of each panel represent a XYZ model of the combination and is analyzed according to Loewe’s model of drug additivity. At the right of each graph is the matrix of combinations that shows the differences between the theoretical combinations and empirical data by a number generated for each point. Positive numbers are given for synergistic combinations, whereas negative numbers indicate antagonism. Color code indicates statistical significance of the difference between theorical and empirical model (Student’s t-test). *p < 0.05. The graphs show the mean ± standard deviation of three independent experiments

Finally, we performed combination assays with O-EO and fluconazole or nystatin and evaluated viability using the MTT reduction method, both for planktonic cells and *C. albicans-*strain biofilms, to determine whether O-EO could potentiate the effects of conventional antifungals. Combinations were performed on 96-well plates with the checkerboard method, using various concentrations of each treatment. To evaluate whether combination effects were antagonistic, additive or synergistic, we used the Loewe synergy model. As shown in Fig. 5, the combinations mainly showed additive effects in planktonic cultures for both strains assayed, with some combinations showing synergistic interactions. For example, in ATCC 90029, some combinations of fluconazole and O-EO exerted a significant synergistic effect in the range of 2–5 mg/L for fluconazole and 0.018–0.135 mg/L for O-EO. In the remaining strains, synergistic effects were less pronounced and were restricted to a few combinations.

The combination assays showed that in the ATCC 90029 strain biofilm, there was a clear synergy between O-EO and both fluconazole and nystatin after 24 h. The heatmaps (ATCC 90029, left) for both drugs showed that most combinations appeared to be synergistic. Indeed, the combination matrix (ATCC 90029, right) confirms that for fluconazole, all combinations with O-EO except one were synergistic (*p* < 0.05). Combinations of O-EO and nystatin also showed several synergistic interactions.

**Fig. 6:**
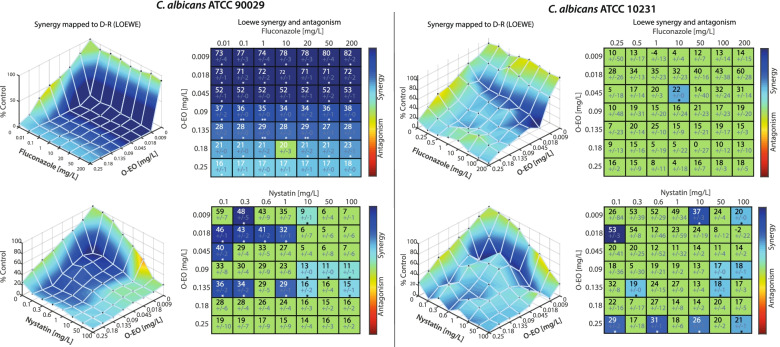
Effect of *Origanum vulgare* essential oil combined with fluconazole and nystatin on the viability of biofilms. The effects of the combination of O-EO with fluconazole (upper row) and nystatin (lower row) on cell viability were measured using the MTT reduction assay in two *C. albicans strains*: ATCC 90029 (left panels) and ATCC 10231 (right panels). Preformed biofilms (24 h) were exposed to the different drugs for 24 h. Seven concentrations were mixed in every possible combination to obtain an effect matrix plotted with the Combenefit software. The graphs at the left of each panel represent a XYZ model of the combination and is analyzed according to Loewe’s model of drug additivity. At the right of each graph is the matrix of combinations that shows the differences between the theoretical combinations and empirical data by a number generated for each point. Positive numbers are given for synergistic combinations, whereas negative numbers indicate antagonism. Color code indicates statistical significance of the difference between theorical and empirical model (Student’s t-test). *p < 0.05. The graphs show the mean ± standard deviation of three independent experiments

In the *C. albicans* ATCC 10231 strain (Fig. 6, upper right panel), the combination of O-EO and fluconazole appeared synergistic at various points in the heatmap; however, statistical analysis showed that most of these interactions were not significant. Combinations of nystatin and O-EO against this strain (Fig. 6, lower right panel) showed results similar to those for ATCC 90029 strain, with synergy apparent at many points in the heatmap. These synergistic interactions could be due to the inhibitory effects of O-EO on adhesion, morphogenesis and biofilm formation. We obtained similar results on oral clinical isolates of *C. albicans* (Supplementary Fig. 2). For further characterize the pharmacodynamic interaction between O-EO and fluconazole and nystatin, we calculated the Fractional Inhibitory Concentration Index (FICI), obtaining synergistic interactions for ATCC90029 strain and the clinical isolates tested. For ATCC 10231 the FICI shows an additive interaction for O-EO and nystatin combination (Supplementary Table 1).

One of the major concerns for the use of essential oils on medicine is their cytotoxicity profile on human cells. We present a preliminary study on Hep G2 cells. O-EO decreased viability of this line derived from human hepatocyte carcinoma by using a Resazurin Cell Viability Kit (Cell Signaling). Hep G2 cells were incubated with _1/2_IC_50_, IC_50_ and 2xIC_50_ for 24 h at 37 °C. Results show that at these concentrations viability of the cells decreased significantly respect to untreated controls (Supplementary Fig. 3).

## Discussion

It is well known that compositions of essential oils from *O. vulgare* and other species from the genus *Origanum* may vary widely due to geographic factors, light conditions and harvesting season [27, 28]. Importantly, several studies have associated the biological activities of essential oils with the presence of specific molecules among their constituents. It has been reported that essential oils containing a higher proportion of oxygenated monoterpenes with respect to monoterpene hydrocarbons show greater antimicrobial activity [29]. Vale-Silva et al. (2011) reported a potent anticandidal effect of essential oil from an *O. vulgare* subspecies, suggesting that this effect could be related to the presence of carvacrol among its main constituents [30]. In a similar study, Bhat et al. (2018) also linked the antifungal effect of *O. vulgare* essential oil to carvacrol [31]. Our results indicate that carvacrol is not among the main constituents of the essential oil analyzed here, which could be attributable to the geographic differences mentioned above. Therefore, other compounds must contribute to the anticandidal effect observed. Candidates include γ-terpinene and thymol, which accounted for 11.66 and 14.20% of the total content, respectively. Consistent with these results, other authors have reported anticandidal activity for *O. vulgare* essential oils with high thymol and γ-terpinene content [32, 33].

Several authors have studied the antifungal activity of *O. vulgare* with variable results, with research focusing mainly on the ability of this substance to inhibit the growth of fungal organisms, using disc diffusion assays and various methods of MIC determination. MIC values ranging from 0.024 mg/L to 300 mg/L against *C. albicans* and non-*albicans* strains have bene reported [30, 31, 34–40].

These data are consistent with our results, as we reported MIC values of 0.01 and 0.97 mg/L in *C. albicans* ATCC 90029 and ATCC 10231 strains, respectively. In non-*albicans* strains, we showed MICs of 5.33 mg/L in *C. krusei* and 2.61 mg/L in *C. dubliniensis*. *C. krusei* and *C. dubliniensis* are not among the most frequent non-*albicans Candida* opportunistic pathogens [41]. However, *C. krusei* is well known for its strong resistance to fluconazole and other antifungals. Furthermore, C*. dubliniensis* is commonly isolated from HIV-infected patients and patients with other emerging fungal infections. Therefore, these results could be useful in developing wide-spectrum anticandidal treatments [7, 42]. This is in agreement with recent reports that propose essential oils from different plants to overcome the high resistance to conventional antifungals in nosocomial infections related to *Candida* spp. [43, 44].

Since the major virulence factors of *Candida* spp. include the ability to adhere to surfaces, alter its morphology and form biofilms, it is important to evaluate not only the antifungal actions of essential oils against planktonic cultures, but also their effectiveness against these three processes. Prior studies have reported antibiofilm effects of essential oils from *Lavandula dentata* L., *Melaleuca alternifolia* M., *Laurus nobilis* L., *Salvia officinalis* L. and other plants, as well as their isolated compounds (terpenes), against *Candida* spp. [17, 18, 21, 45–47]. However, in the case of *O. vulgare*, most prior studies addressed antifungal activity against planktonic cultures of fungal cells, while analyses of potential antibiofilm effects were mainly focused on bacterial microorganisms [48]. Therefore, one of the main objectives of this study was to describe the antivirulence and antibiofilm properties of O-EO against *Candida* spp. beyond the classical antifungal effects. Interestingly, O-EO inhibited morphogenesis not only in *C. albicans* but also in *C. krusei* and *C. dubliniensis* strains (Fig. 2). This effect on morphogenesis is relevant, as it is well accepted that morphogenesis is linked to virulence and antifungal drug susceptibility [15]. Adhesion is another virulence trait that not only explains the versatility of *Candida* spp. in colonizing abiotic and biological niches, but also explains, at least in part, the species’ antifungal resistance [49]. O-EO significantly inhibited adhesion in *C. albicans* strains at the same concentration that inhibited growth of planktonic cultures (Fig. 3). Although there are no reports of O-EO affecting adhesion in *C. albicans*, it has been described that 4-terpineol and thymol have anti-adhesion effects [45, 50]. In addition, 4-terpineol can inhibit the viability and structural arrangement of *C. albicans* biofilms [51, 52]. Thymol has shown similar effects against *C. albicans* and *C. tropicalis* biofilms, affecting both viability and membrane integrity [53, 54]. Nevertheless, these studies were conducted with essential oils from other aromatic plant species. Our biofilm scratch assay showed that O-EO inhibited biofilm proliferation. However, these results may be also related to inhibition of morphogenesis and adhesion by O-EO, making it difficult for the established biofilm to recolonize the area where the cells were removed mechanically. It is important to note that the biofilm scratch assay results were observed at concentrations lower than the IC_50_ obtained for biofilm viability.

Although we cannot completely rule out the possibility that the inhibition of virulence patterns might be also associated with its actions on cell viability, it is important to note that we tested the effect of O-EO using the MIC obtained for planktonic budding yeast against filamentous cells, the adhesion process and biofilm formation, at concentrations almost three orders of magnitude lower than the IC_50_ value that inhibited biofilm viability (Table 3). Thus, the observed effect of the O-EO is mainly related to inhibition of virulence processes rather than impaired viability. Several reports have shown that antivirulence drugs against *Candida* spp. are often associated with modulation of genes encoding for proteins involved in positive regulation of biofilm formation, such as *HWP1, ALS3* and *FKS1* [9, 55]. Most studies that have described the antibiofilm properties of essential oils against *Candida* spp. have not reported the molecular mechanisms behind their observed effects, but there are reports that have correlated the antivirulence actions of essential oils from various plant specimens to modulation of the expression of several genes associated with filamentation, adhesion and biofilm formation. Through RT-PCR analysis, Khan et al. (2014) showed that *Ocimum sanctum* essential oil reduced expression of the *HWP1, SAP1* and *PLB2* genes, although the exact composition of the essential oil tested was not reported [56]. Similarly, isolated compounds from cedar leaf essential oil (*Cedrus libani*) downregulated the *ECE1, ECE2, RBT1* and *EED1* genes, which are associated with biofilms. The complete essential oil, however, was not evaluated [57]. Another recent report showed that essential oil obtained from *Mentha piperita* (peppermint) downregulated the expression of hyphal wall protein 1 gene (*HWP1*), but its main constituents were not reported [58]. Although our results on virulence patterns could be related to gene expression inhibition in *Candida* spp., this idea must be validated using RT-PCR.

There have been studies reporting the combination effect of essential oils with conventional antifungals. Most of these studies have demonstrated synergy between essential oils and azoles and polyenes against susceptible and resistant *Candida* spp. strains and fungi from other species in their planktonic forms [19, 59–61]. In the present study, O-EO showed a synergistic effect with fluconazole against the *C. albicans* ATCC 90029 ant ATCC 10231 strains, while the combination effect with nystatin was less evident and was only present at the highest concentrations. Notably, in the biofilm assays, there were also synergistic interactions between O-EO and fluconazole and nystatin, for both strains assayed, with especially striking results in the ATCC 90029 strain. The presence of diverse molecules in essential oil may enhance the prospects of these substances as antimicrobials, as the various components could act simultaneously against different pharmacological targets, increasing their spectrum in comparison with traditional antifungals.

*O. vulgare* essential oil has shown cytotoxic effects on normal and tumor cell lines [62]. We made a preliminary assay to evaluate the cytotoxicity of O-EO on Hep G2 cells, showing a high cytotoxicity for this cancer cell line (Supplementary Fig. 2). This raises concern for the development of antimicrobial strategies based on this essential oil. One approach to overcome this problem is the use of essential oil in combination with classic antifungals, since their synergistic interactions observed in this study would allow for lower doses, thereby mitigating toxicity. Further studies are required to validate this hypothesis in normal human cell lines.

## Conclusion

Given the lack of studies evaluating O-EO in combination with classic antifungals against *C. albicans* biofilms, the results of the present study are promising. Although the safety and toxicity of essential oils must be addressed in more complex scenarios, such as in vivo models, the present study in fact suggests that use of *O. vulgare* essential oil should be considered as a strategy to combat virulence patterns and potentiate the effects of antifungals. We propose that synergistic interactions could be a consequence of the effects of O-EO on these biofilm-related processes. Future studies focusing the properties of isolated compounds are needed to better understand the underlying mechanisms of this extract against virulence patterns in *Candida* spp. Finally, it will be important to develop new formulations that provide a sustained release of essential oil components, at lower concentrations, to avoid toxicity. In addition, it is necessary to evaluate combinations with other clinically useful antifungals such as clotrimazole, ketoconazole and miconazole, which are indicated for topical treatment. We are currently working in these directions for the treatment of oral candidiasis.

## Supplementary Information

**Additional file 1: Supplementary Figure 1**. GC-MS chromatogram of essential oil of *O. vulgare*. Letters indicate the major constituents identified in the sample. a: γ-terpinene; b: cis-sabinene hydrate; c: 4-terpineol; d: thymol.

**Additional file 2: Supplementary Figure 2**. Effect of *Origanum vulgare* essential oil combined with fluconazole and nystatin on the viability of biofilms from *Candida albicans* clinical isolates. The effects of the combination of O-EO with fluconazole (upper row) and nystatin (lower row) on cell viability were measured using the MTT reduction assay in two *C. albicans* clinical isolates from denture stomatitis patients: 17p (left panels) and 18r (right panels). Preformed biofilms (24h) were exposed to the different drugs for 24h. Seven concentrations were mixed in every possible combination to obtain an effect matrix plotted with the Combenefit software. The graphs at the left of each panel represent a XYZ model of the combination and is analyzed according to Loewe’s model of drug additivity. At the right of each graph is the matrix of combinations that shows the differences between the theoretical combinations and empirical data by a number generated for each point. Positive numbers are given for synergistic combinations, whereas negative numbers indicate antagonism. Color code indicates statistical significance of the difference between theorical and empirical model (Student’s t-test). *p < 0.05. The matrix of combinations shows the mean ± standard deviation of three independent experiments.

**Additional file 3: Supplementary Figure 3**. Cytotoxicity of O-EO on Hep G2 cells. Cell viability, measured as values of resazurin reduction test in relative fluorescence units (RFU), for Hep G2 cells incubated with oregano oil at ½ IC_50_; IC_50_ and 2xIC_50_ obtained for biofilms. H_2_O_2_ was used as a positive control for cytotoxicity. Cells (1 × 10^5^ cells) were seeded in 96-well flat bottom plates (Falcon) in 100 μL of Dulbecco’s Modified Eagle Medium (DMEM) containing 10% FBS and 1% penicillin-streptomycin in absence or presence of different treatments. Then culture cells were incubated in a humidified incubator for 24 h at 37 °C and 5% CO2. After this period, viability of the cells was evaluated using a resazurin based kit according to manufacturer’s instructions (Sigma-Aldrich, Darmstadt, Germany; Cat No. TOX8-1KT). Bars show the mean results from three independent experiments. Error bars denote SD; ****P < 0.0001 compared with control (medium).

**Additional file 4: Supplementary Table 1**: Mean Fractional Inhibitory Concentration Index (FICI) of O-EO in combination with fluconazole or nystatin against *C. albicans* biofilms.

**Additional file 5.** Supporting Information. Certificate of identification of O. vulgare plant material.

## Data Availability

All data generated or analyzed during this study are included in this published article.
