# Supplementary material for: Origanum vulgare L. essential oil inhibits virulence patterns of Candida spp. and potentiates the effects of fluconazole and nystatin in vitro
Source: BMC Complement Med Ther. 2022 Feb 9;22:39. doi: 10.1186/s12906-022-03518-z (PMC8827202; doi:10.1186/s12906-022-03518-z)
Supplement: Supplementary file 1 — Additional file 1: Supplementary Figure 1. GC-MS chromatogram of essential oil of O. vulgare. Letters indicate the major constituents identified in the sample. a: γ-terpinene; b: cis-sabinene hydrate; c: 4-terpineol; d: thymol. [file 12906_2022_3518_MOESM1_ESM.pdf]

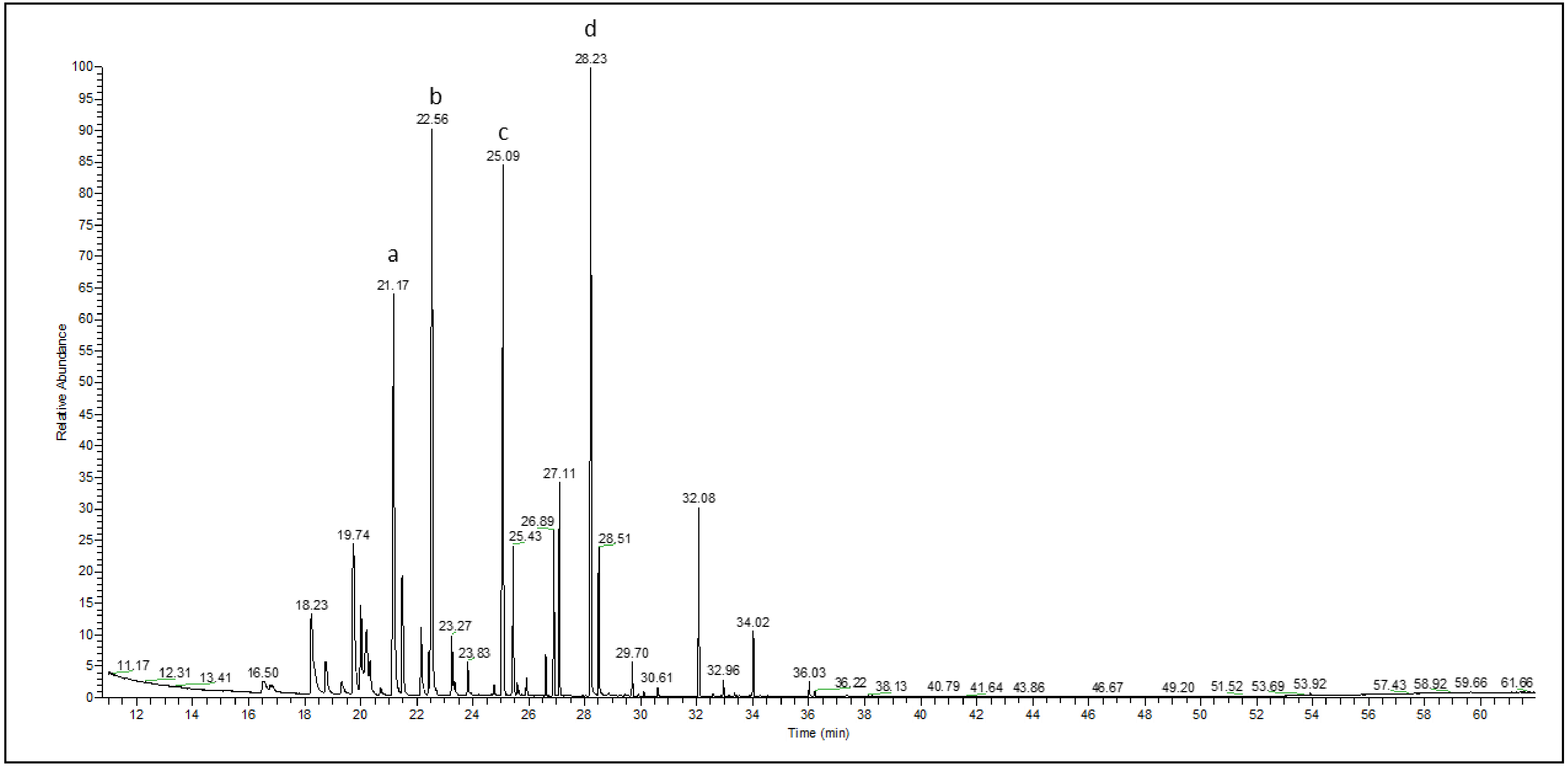

Fig. S1 GC-MS chromatogram of essential oil of *O. vulgare.* Letter indicate the major constituents identified in the sample. a: γ-terpinene; b: *cis*-sabinene hydrate; c: 4-terpineol; d: thymol.
