# Supplementary figures and images for: Origanum vulgare L. essential oil inhibits virulence patterns of Candida spp. and potentiates the effects of fluconazole and nystatin in vitro
Source: BMC Complement Med Ther. 2022 Feb 9;22:39. doi: 10.1186/s12906-022-03518-z (PMC8827202; doi:10.1186/s12906-022-03518-z)

*C. albicans* 17p

Fluconazole

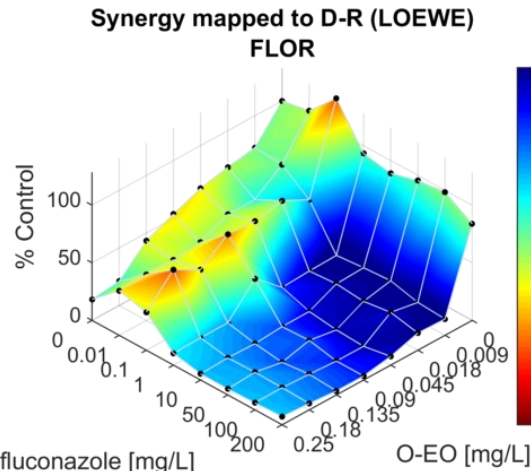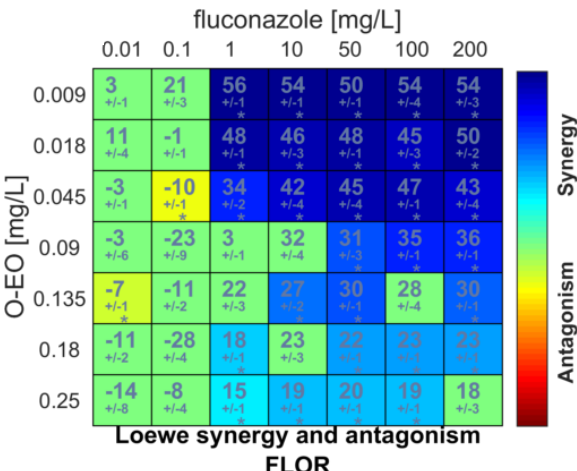

Nystatin

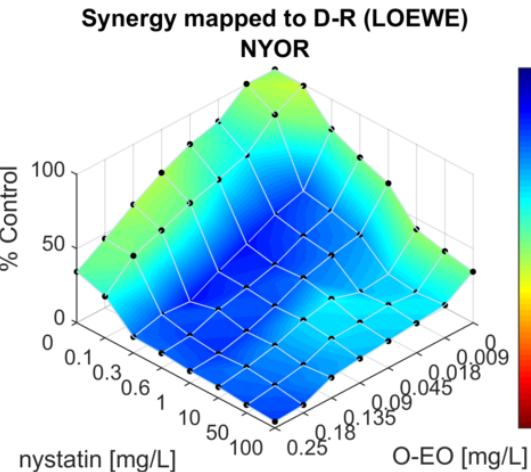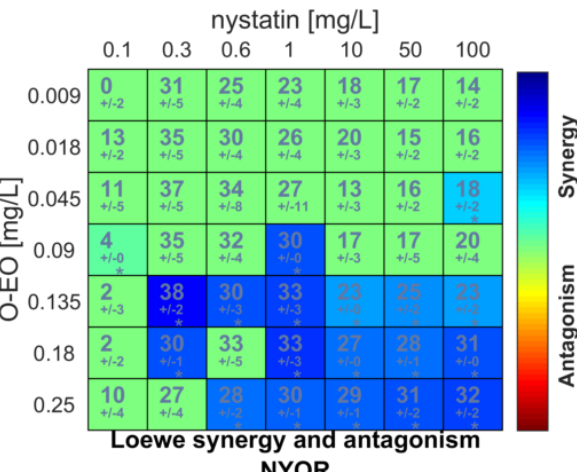

*C. albicans* 18r

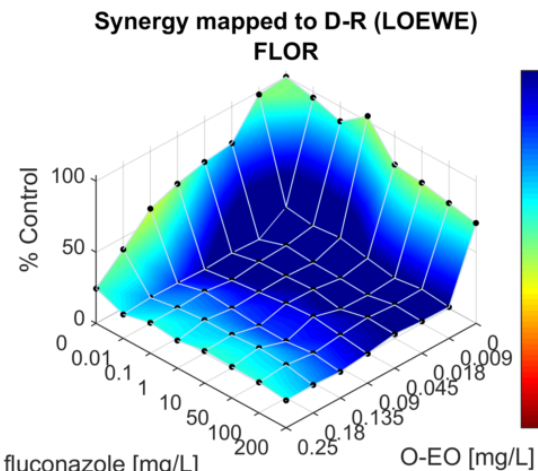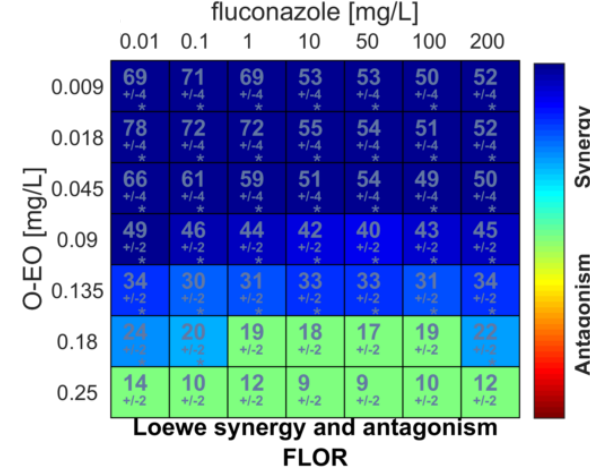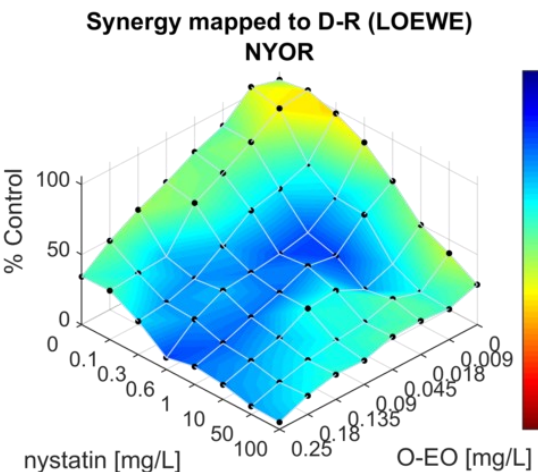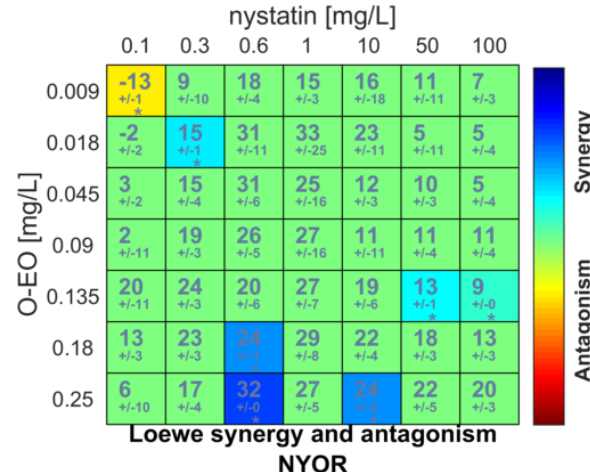

Supplement: Supplementary file 2 — Additional file 2: Supplementary Figure 2. Effect of Origanum vulgare essential oil combined with fluconazole and nystatin on the viability of biofilms from Candida albicans clinical isolates. The effects of the combination of O-EO with fluconazole (upper row) and nystatin (lower row) on cell viability were measured using the MTT reduction assay in two C. albicans clinical isolates from denture stomatitis patients: 17p (left panels) and 18r (right panels). Preformed biofilms (24h) were exposed to the different drugs for 24h. Seven concentrations were mixed in every possible combination to obtain an effect matrix plotted with the Combenefit software. The graphs at the left of each panel represent a XYZ model of the combination and is analyzed according to Loewe’s model of drug additivity. At the right of each graph is the matrix of combinations that shows the differences between the theoretical combinations and empirical data by a number generated for each point. Positive numbers are given for synergistic combinations, whereas negative numbers indicate antagonism. Color code indicates statistical significance of the difference between theorical and empirical model (Student’s t-test). *p < 0.05. The matrix of combinations shows the mean ± standard deviation of three independent experiments. [file 12906_2022_3518_MOESM2_ESM.pdf]

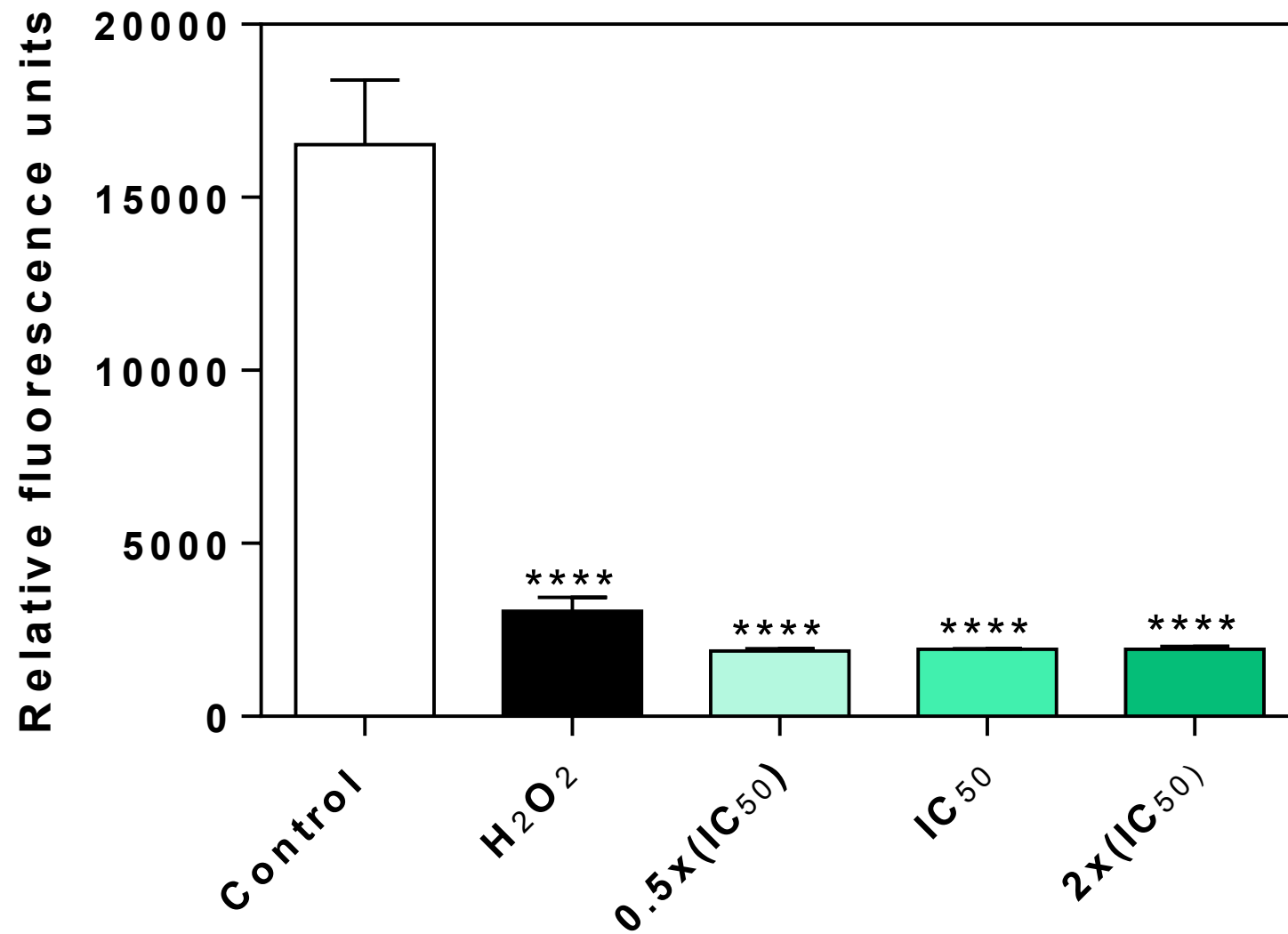

Supplement: Supplementary file 3 — Additional file 3: Supplementary Figure 3. Cytotoxicity of O-EO on Hep G2 cells. Cell viability, measured as values of resazurin reduction test in relative fluorescence units (RFU), for Hep G2 cells incubated with oregano oil at ½ IC50; IC50 and 2xIC50 obtained for biofilms. H2O2 was used as a positive control for cytotoxicity. Cells (1 × 10^5^ cells) were seeded in 96-well flat bottom plates (Falcon) in 100 μL of Dulbecco’s Modified Eagle Medium (DMEM) containing 10% FBS and 1% penicillin-streptomycin in absence or presence of different treatments. Then culture cells were incubated in a humidified incubator for 24 h at 37 °C and 5% CO2. After this period, viability of the cells was evaluated using a resazurin based kit according to manufacturer’s instructions (Sigma-Aldrich, Darmstadt, Germany; Cat No. TOX8-1KT). Bars show the mean results from three independent experiments. Error bars denote SD; ****P < 0.0001 compared with control (medium). [file 12906_2022_3518_MOESM3_ESM.pdf]
