# Supplementary material for: Origanum vulgare L. essential oil inhibits virulence patterns of Candida spp. and potentiates the effects of fluconazole and nystatin in vitro
Source: BMC Complement Med Ther. 2022 Feb 9;22:39. doi: 10.1186/s12906-022-03518-z (PMC8827202; doi:10.1186/s12906-022-03518-z)
Supplement: Supplementary file 4 — Additional file 4: Supplementary Table 1: Mean Fractional Inhibitory Concentration Index (FICI) of O-EO in combination with fluconazole or nystatin against C. albicans biofilms. [file 12906_2022_3518_MOESM4_ESM.docx]

**Supplementary table 1:** Mean Fractional Inhibitory Concentration Index (FICI) of O-EO in combination with fluconazole or nystatin against *C. albicans* biofilms.

|  | **ATCC 90029** | | **ATCC 10231** | | **17p** | | **18r** | |
| --- | --- | --- | --- | --- | --- | --- | --- | --- |
| **Combination** | FICI | Effect | FICI | Effect | FICI | Effect | FICI | Effect |
| O-EO / FCZ | 0.1 | Synergistic | 0.4 | Synergistic | 0.3 | Synergistic | 0.1 | Synergistic |
| O-EO/ Nys | 0.1 | Synergistic | 0.6 | Additive | 0.4 | Synergistic | 0.4 | Synergistic |

Fractional inhibitory Concentration Index was calculated by using the following equation: FICI = FIC_A_ + FIC_B_, where FIC_A_ = MIC of the combination/MIC_A_ alone; FIC_B_ = MIC of the combination/MIC_B_ alone. The FICI was interpreted as follows: (1) a synergistic effect when FICI ≤0.5; (2) an additive or indifferent effect when FICI >0.5 and <1 and (3) an antagonistic effect when FICI >1.
