# Supplementary material for: Origanum vulgare L. essential oil inhibits virulence patterns of Candida spp. and potentiates the effects of fluconazole and nystatin in vitro
Source: BMC Complement Med Ther. 2022 Feb 9;22:39. doi: 10.1186/s12906-022-03518-z (PMC8827202; doi:10.1186/s12906-022-03518-z)
Supplement: Supplementary file 5 — Additional file 5. Supporting Information. Certificate of identification of O. vulgare plant material. [file 12906_2022_3518_MOESM5_ESM.pdf]

HERBARIO CONC
Facultad de Ciencias Naturales y Oceanográficas
UNIVERSIDAD DE CONCEPCIÓN-CHILE
Casilla 160-C, Teléfonos: 56-41-2204418/2204967, Fax: 56-41-2246005

Dr. Mario Diaz-Dosque
Instituto de Investigación en Ciencias Odontológicas
Facultad de Odontología
Universidad de Chile

Concepción, 12 de octubre de 2021

Estimado Dr. Diaz-Dosque,

Respecto de la muestra vegetal analizada para su confirmación taxonómica, espécimen proveniente de Chicauma, Comuna de Lampa, Provincia de Chacabuco, Región Metropolitana, se consultó literatura especializada, y se comparó con ejemplares de herbario, con esto se llegó a confirmar que la muestra vegetal corresponde a la especie *Origanum vulgare* L.

El ejemplar se ingresa al herbario con el número CONC 191040.

Sin otro particular, le saluda atentamente,

Alicia Marticorena Garri
Curadora
Herbario CONC
